# Hospitals in India

**Published:** 1893-12-30

**Authors:** 


### HOSPITALS IN INDIA.

### I.—THE BENGAL PRESIDENCY.

(6) Civil Dispensaries and Police Hospitals in Burma.

A.—Lower Burma.

At the end of 1891 there were 35 dispensaries in Lower Burma, with accommodation for 1,016 in-patients. The number of in-patients treated was 20,028, of whom 11,821 were cured, 1,072 were relieved, 1,511 were discharged otherwise, and 1,248 died. The daily average number of beds occupied was 680, and the percentage of deaths to total treated was only six. Out-patients numbered 264,321, of whom 206,622 attended personally, the others being represented by friends; the daily average attendance at this department was 1,545. Indoor and outdoor patients together thus numbered 284,349, an increase of 21,548 over the previous year. It is extremely satisfactory to note that of the grand total treated no less than 140,172, or 49·30 per cent., were Burmese, which is unmistakable evidence of the popularity of these institutions. During the year under review 337 major surgical operations were performed, with the result that 270 patients were cured, 13 were relieved, 19 were discharged otherwise, and 12 died.

The total income of these dispensaries was Rs.2,30,489, of which sum Government provided Rs.12,262, local funds Rs.66,399, and municipal funds Rs.1,37,563, while the Europeans subscribed Rs.5,016, natives Rs.2,574, and patients paid Rs.5,990. The total expenditure tallied with the total revenue, and the items call for no particular comment. These dispensaries appear to be managed with economy and ability, and have achieved a measure of popularity among the indigenous population which has not been acquired by the dispensaries in any other district in the Presidency.

### B.—Upper Burma.

In Upper Burma 45 dispensaries were in operation on the last day of 1891 with accommodation for 602 indoor patients, the daily average number of beds occupied being 318. During the year 8,575 in-patients were treated, and of these 7,119 were cured, 283 were relieved, 217 were discharged otherwise, and 379 died, the percentage of deaths to total treated being four. Out-patients numbered 127,869, with a daily average attendance of 1,037. Indoor and outdoor patients combined thus numbered 136,444, of whom 84,215 were Burmese, or 61·7 per cent. of the total treated. Upon the treatment and maintenance of all these patients a gross sum of Rs.1,15,990 was expended; the bulk of the cost is borne by the Government, and the subscriptions were very considerably less in Upper than in Lower Burma.

The question of the establishment of hospital committees in Upper Burma is now under consideration. Orders for the formation of committees were issued early in the year, but the precise extent to which funds for the maintenance of hospitals made over to the committees are to be contributed from provincial and municipal funds respectively has not yet been finally settled. Municipal committees in Lower Burma have been urged to transfer the administration of their hospitals to special hospital committees formed by delegates from their own bodies, and by other gentlemen interested in hospital management, and they have been asked to make yearly grants in support of the hospitals, to be supplemented by such contributions from private sources as the hospital committees are able to collect. Under this system the private contributions thus obtained will be spent directly on charitable purposes, and will not be paid into the Treasury.

### C.—Police Hospitals.

The average strength of the military police force in Upper and Lower Burma was 15,160 in 1891, as compared with 17,779 in 1890. The total number of admissions into hospital was 23,323, with a daily average number of 825 sick; sick leave to their homes was granted to 408 men; and 258 deaths occurred, or an average of 17·02 per mille of the strength. The admission rate per mille of strength was 16·65, or 1·3 less than the previous year, and the invaliding rate was 26·91 per mille, or 10·71 less than the previous year. Notwithstanding a slight increase of ·37 in the death-rate, the figures for 1891 are on the whole satisfactory. The gross expenditure on police hospitals was Rs.1,35,726, as compared with Rs.1,54,121 in 1890, the decrease occurring under all heads of account.

